# Pectoralis muscle area and mortality in smokers without airflow obstruction

**DOI:** 10.1186/s12931-018-0771-6

**Published:** 2018-04-10

**Authors:** Alejandro A. Diaz, Carlos H. Martinez, Rola Harmouche, Thomas P. Young, Merry-Lynn McDonald, James C. Ross, Mei Lan Han, Russell Bowler, Barry Make, Elizabeth A. Regan, Edwin K. Silverman, James Crapo, Aladin M. Boriek, Gregory L. Kinney, John E. Hokanson, Raul San Jose Estepar, George R. Washko

**Affiliations:** 1 Division of Pulmonary and Critical Care Medicine, Department of Medicine, Brigham and Women’s Hospital, Harvard Medical School, 75 Francis Street, Boston, MA 02115 USA; 2 Division of Pulmonary & Critical Care Medicine, University of Michigan Health System, Ann Arbor, MI USA; 3 Department of Radiology, Brigham and Women’s Hospital, Harvard Medical School, Boston, MA USA; 4 Division of Pulmonary, Allergy and Critical Care Medicine, University of Alabama at Birmingham, Birmingham, AL USA; 5 Department of Medicine, Division of Pulmonary and Critical Care Medicine, National Jewish Health, Denver, CO USA; 6 Channing Division of Network Medicine, Brigham and Women’s Hospital, Harvard Medical School, Boston, MA USA; 7 Division of Pulmonary and Critical Care Medicine, Baylor College of Medicine, Houston, Texas USA; 8 Colorado School of Public Health, University of Colorado-Denver, Aurora, CO USA

**Keywords:** Pectoralis muscle mass, CT, Smoking, Paravertebral muscle mass

## Abstract

**Background:**

Low muscle mass is associated with increased mortality in the general population but its prognostic value in at-risk smokers, those without expiratory airflow obstruction, is unknown. We aimed to test the hypothesis that reduced muscle mass is associated with increased mortality in at-risk smokers.

**Methods:**

Measures of both pectoralis and paravertebral erector spinae muscle cross-sectional area (PMA and PVMA, respectively) as well as emphysema on chest computed tomography (CT) scans were performed in 3705 current and former at-risk smokers (≥10 pack-years) aged 45–80 years enrolled into the COPDGene Study between 2008 and 2013. Vital status was ascertained through death certificate. The association between low muscle mass and mortality was assessed using Cox regression analysis.

**Results:**

During a median of 6.5 years of follow-up, 212 (5.7%) at-risk smokers died. At-risk smokers in the lowest (vs. highest) sex-specific quartile of PMA but not PVMA had 84% higher risk of death in adjusted models for demographics, smoking, dyspnea, comorbidities, exercise capacity, lung function, emphysema on CT, and coronary artery calcium content (hazard ratio [HR] 1.85 95% Confidence interval [1.14–3.00] *P* = 0.01). Results were consistent when the PMA index (PMA/height^2^) was used instead of quartiles. The association between PMA and death was modified by smoking status (*P* = 0.04). Current smokers had a significantly increased risk of death (lowest vs. highest PMA quartile, HR 2.25 [1.25–4.03] *P* = 0.007) while former smokers did not.

**Conclusions:**

Low muscle mass as measured on chest CT scans is associated with increased mortality in current smokers without airflow obstruction.

**Trial registration:**

NCT00608764

**Electronic supplementary material:**

The online version of this article (10.1186/s12931-018-0771-6) contains supplementary material, which is available to authorized users.

## Background

There are an estimated 40 million current smokers in the United States [1] and such noxious exposure is linked to cancer, cardiovascular disease, and respiratory conditions such as chronic obstructive pulmonary disease (COPD). Recent investigation suggests that even smokers without expiratory airflow obstruction experience symptomatic impairment and decrements in health status and exercise capacity [2, 3]. Further, smokers without airflow obstruction (hereafter referred to as at-risk smokers) present changes in skeletal muscle structure and have low muscle mass similar to those with COPD [4–8]. Low muscle mass is frequent and associated with increased risk for death in the general population [9–12] and those with COPD [13–17] and cancer [18]. It is unclear, however, if low skeletal muscle mass in at-risk smokers has prognostic value. Such knowledge would provide new insight into the multiple comorbidities present in those susceptible to injury from chronic tobacco smoke exposure, which in turn could result in improved clinical care.

A variety of techniques such as bioimpedance and dual energy X-ray absorbance have been used to assess fat-free mass, a surrogate for muscle mass [14, 19–21]. These assessments are, however, holistic and cannot discriminate muscle-specific changes or their unique clinical significance. Such efforts have been undertaken using computed tomographic (CT) imaging. A myriad of studies has demonstrated that the CT cross-sectional area of mid-thigh, pectoralis, and paravertebral erector spinae muscles are associated with fat-free mass, handgrip strength, exercise capacity, health status, and COPD severity [22–25]. These CT measures were also predictive of mortality in subjects with established COPD [23, 24, 26]. Additionally, CT allows measuring fat depots including subcutaneous adipose tissue and thus provides additional prognostic understanding of the body composition [27].

Based on prior studies, [15, 23, 24] we hypothesized that CT measures of body composition including distinct skeletal muscle groups would differentially predict death in at-risk smokers. To test this hypothesis, we examined the CT and clinical data obtained in the COPDGene Study [28].

## Methods

**Fig. 1:**
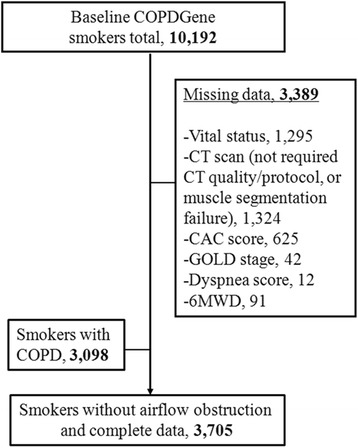
Flowchart showing the subjects selection

The institutional review board of each of the 21 participating centers approved the study and all participants gave written informed consent. The Partners HealthCare Research Committee approved the current analysis (2007P-000554). Methodological details are provided in this section as well as in the online supplement. Briefly, COPDGene enrolled 10,192 Non-Hispanic white and African-American current and former smokers (10 or more pack-years) who were 45–80 years old between 2008 and 2013 [28]. Smokers with active lung diseases other than COPD, emphysema, or asthma were excluded. In this analysis, we used smokers who did not meet criteria for COPD as defined by spirometry (see below) and had complete data on mortality, muscle measurements and other relevant covariates (Fig. 1). Data on PMA and mortality in smokers with COPD was presented as part of a separate report [26].

### COPDGene imaging protocol

Chest CT images without the administration of intravenous iodinated contrast were obtained with subjects in the supine position at both full inflation (approximating total lung capacity) and relaxed exhalation (approximating functional residual capacity). Acquisition parameters included: 120 kVp, 200 mAs, and 0.5 rotation time. Images were reconstructed using the following filters, slice thicknesses and spacing: standard, B31f, and B kernel; 0.625, 0.75, and 0.90 mm slice thickness; 0.625, 0.5, and 0.45 mm slice interval for General Electric Medical System, Siemens, and Philips scanners, respectively [29]. Data collected from the inspiratory CT scans included densitometric measures of the parenchyma, body composition (pectoralis muscle area [PMA], paravertebral erector spinae muscle area [PVMA], and subcutaneous adipose tissue [SAT]), and coronary artery calcium (CAC) content. Emphysema-like tissue was defined as percentage of low attenuation areas below − 950 (%LAA-950) Hounsfield units (HU) evident on the inspiratory CT scans and was assessed using the Chest Imaging Platform (www.chestimagingplatform.org) [30].

### CT muscle measurements

The PMA, PVMA, and SAT measurements were performed on inspiratory CT scans by trained analysts who were blinded to subjects’ data [22, 27]. For the PMA, the analyst visually identified the superior aspect of the aortic arch and then the first axial slice above the arch. This location was chosen because it was easy to identify and could be replicated across a large cohort of participants. The left and right pectoralis major and minor muscles were then identified on the anterior chest and their edges manually segmented using a pre-defined attenuation range of − 50 and 90 HU. The intra- and inter-analyst concordance correlation coefficients (CCC) for PMA were 1.00 to 0.98, respectively [27]. The PVMA was measured on a single-axial slice at the level of spinous process of the first lumbar vertebrae using the same HU thresholds utilized for PMA. A subset of 19 subjects was used to measure the intra- and inter-analyst agreement of PVMA. The SAT was defined as the region of interest between the pectoralis major muscles and skin surface on those same axial slices used to measure PMA and their edges were manually determined using a range of − 200 and 0 HU. The intra- and inter-analyst CCC for SAT were 1.00 and 0.99, respectively [27]. We used this limited subcutaneous fat region because the entire circumference of the chest was not available in many CT scans. As the focus of this investigation was the effect of low muscle mass on mortality, PMA, PVMA, and SAT were divided in 4 groups using the sex-specific quartile cut-off points with the quartile with the highest cross sectional area (4th) acting as the referent subgroup for the main analyses. We used sex-specific quartiles because of marked sex-based differences in PMA [27]. In a secondary analysis, the PMA was also expressed as an index (muscle cross-sectional area/height^2^) to account for between-subject differences in body size where lower index values indicate lower muscle mass.

### Coronary artery calcium assessment

Previous investigation of the COPDGene data demonstrated a strong correlation between CAC measured on non-cardiac- and electrocardiographically-gated (cardiac) CT scans (*r* = 0.96) [31]. Coronary calcium was identified in 3 contiguous voxels by using a cutoff point of 130 HU resulting in a minimum calcified lesion area of 1.02 mm^2^ and a lesion score was calculated as the product of lesion area and CT attenuation. The attenuation factor is derived from the maximal HU within the area as described by Agatston, ranging from 1 to 4. A total CAC score was obtained by aggregating individual lesion scores from each of 4 anatomic sites (left main, left anterior descending, circumflex, and right coronary arteries). CAC has been used to detect subclinical atherosclerosis [32, 33] and details of coronary artery calcium assessment have been described elsewhere [31, 34, 35]. A subject’s CAC score of 0 Agatston Unit indicates absence of calcium. Because CAC scores are not normally distributed, it was log transformed.

### Clinical and lung function assessments

Clinical data including eight comorbidities and medication use were collected with standardized questionnaires, which are available at www.copdgene.org. Comorbidities including congestive heart failure, stroke, gastroesophageal reflux, cancer, diabetes mellitus, hypertension, coronary artery disease, and asthma were coded based on combining self-reported physician diagnosed conditions and medication use for these conditions. If the participant responded yes to the following question: “Have you ever been told by a physician that you have [condition name]?” was considered to have congestive heart failure, stroke, gastroesophageal reflux, and cancer. Additionally, diabetes and hypertension were considered present if the participant had responded yes to the above question and he/she was receiving treatment for those conditions. Positive responses about heart attack, angina, and coronary artery disease as well as self-report history of angioplasty, cardiac stent, or coronary artery bypass surgery were combined into one condition defined as coronary artery disease. Participants were considered to have had asthma if they responded yes to all the following questions: “Have you ever had asthma?”, “Was it diagnosed by a doctor or other health professional?” “Do you still have it?” [36] Comorbidities were grouped as 0, 1–2, and 3 or more [37]. Body mass index (BMI) measures were also collected at baseline [38]. Dyspnea was evaluated using the modified Medical Research Council (MMRC) Dyspnea score. Exercise capacity was measured using the six-minute walk distance (6MWD) test [39]. Spirometric measures of lung function were conducted following the American Thoracic Society recommendations and the post-bronchodilator forced expiratory volume in one second (FEV_1_) and forced vital capacity (FVC) were expressed as percent of predicted values [40, 41]. Ever smokers whose FEV_1_/FVC ratio was ≥0.7 were defined as “at-risk” smokers [42].

### Mortality ascertainment

Vital status ascertainment was performed through the Social Security Death Index (SSDI) and the COPDGene longitudinal follow-up (LFU) program. Vital status for those who were searched via SSDI were back censored by 3 months to account for expected lag time between a death and subsequent annotation in the SSDI dataset. Vital status for those in whom consistent follow-up was supplied via the LFU program was censored on April 15th, 2015. For LFU participants who died prior to this date, the death was used if an LFU contact was performed in the previous six months. The median follow-up was 6.5 years. We used all-cause mortality as the outcome of interest.

### Statistical analysis

Data are presented as mean ± SD, numbers, and proportion (%). We performed Kaplan-Meier cumulative survival probability plots by sex-based quartiles of PMA, PVMA, and SAT. The log rank test was used to compare survival across groups. Multivariable Cox proportional hazards models were used to assess the association between measures of body composition and mortality. The body composition measurements that were statistically significant in univariate Kaplan-Meier analysis were used for multivariable modeling. Covariates included age, sex, race, height (to account for differences in body size), body mass index ([BMI] categories, < 20, 20–24.9, 25–25.9, ≥30), current smoking status, pack-years of smoking (per 10), dyspnea score (categories, 0–1, ≥2), the number of comorbidities (categories, 0, 1–2, ≥3), 6MWD (per 30 m), FEV_1_, log %LAA-950 on CT, log CAC score, and scanner brand/make. Interactions between PMA and age, sex, race, and smoking status (current and former) were also tested. The final models were checked to meet hazard proportionality assumptions. The intra- and inter-analyst agreement assessment for PVMA was performed using the CCC. Analyses were performed with SAS 9.4 (SAS Institute, Cary NC) using an alpha-error value of 0.05.

## Results

The intra- and inter-analyst CCC for PVMA was 0.964 and 0.947, respectively. Complete clinical, CT, and vital status data were available in 3705 at-risk ever smokers (Fig. 1). PMA and PVMA were significantly correlated (R^2^ = 0.44 *P* < 0.0001) each other (Additional file 1: Figure S1**)**. The baseline characteristics of the cohort are provided by PMA quartile in Table 1 and by PVMA quartile (Additional file 1: Table S1). Those in the lowest (vs. highest) quartiles of PMA were more likely to be older, female, non-Hispanic white and former smokers. These subjects also were shorter, and had lower BMI and CAC score as well as higher %LAA-950 on CT scan. The FEV_1_% predicted, FVC% predicted, and 6MWD were similar between these two groups. When examined by PVMA quartile (Additional file 1: Table S1) the trends were similar to those of PMA quartiles but for %LAA-950. %LAA-950 was not significantly different between the lowest and highest PVMA quartiles. In the overall cohort, there were substantial differences in PMA and PVMA between men and women (54.7 ± 16.6 vs. 34.0 ± 9.8 cm^2^, *P* < .0001), which is the basis for conducting the main analyses using sex-specific quartiles of these CT metrics.

**Table 1:** Baseline characteristics of at-risk smokers by quartile of PMA (*N* = 3705)

	PMA Quartile^a^							
Variable	1 (< 31.8 cm^2^)		2 (31.8–41.2 cm^2^)		3 (41.3–53.5 cm^2^)		4 (> 53.5 cm^2^)	
Age, −yrs	61	± 9	58	± 8	57	± 8	54	± 7
Male Sex, −%	6		33		70		90	
Non-Hispanic White, −%	87		74		66		42	
Height, −cm	163	± 7	168	± 9	173	± 9	176	± 8
BMI, −kg/m2	27	± 5	29	± 6	30	± 6	31	± 6
Pack Years of Smoking	37	± 20	39	± 22	40	± 21	38	± 20
Current Smoking Status, −%	42		54		59		65	
Modified Medical Research Council Dyspnea Score > 1, −%	21		29		27		27	
No. of Comorbidities^a^, −%								
0	39		37		40		45	
1–2	51		51		48		45	
3 or more	10		12		12		10	
FEV_1_, −% predicted	93	± 15	91	± 15	92	± 16	92	± 16
FVC, −% predicted	93	± 14	91	± 14	91	± 15	91	± 16
Six-minute walk distance, −m	455	± 100	443	± 111	452	± 114	456	± 109
PMA, −cm^2^	26.2	± 3.9	36.4	± 2.7	46.9	± 3.6	67.6	± 14.1
PVMA, −cm^2^	39.8	± 7.4	47.7	± 9.9	56.1	± 10.7	64.5	± 12.2
SAT, −cm^2^	67.9	± 32.7	69.4	± 37.2	58.7	± 38.0	51.7	± 37.7
%LAA-950 on CT scans, −%	2.1	± 2.8	2.0	± 2.9	1.9	± 2.2	1.2	± 1.6
CAC score	82	± 210	128	± 339	140	± 309	105	± 289

Data are presented as number, proportion (percentage), and mean ± SD

^a^PMA quartiles are not sex-specific. Missing data for SAT, 44

**Fig. 2:**
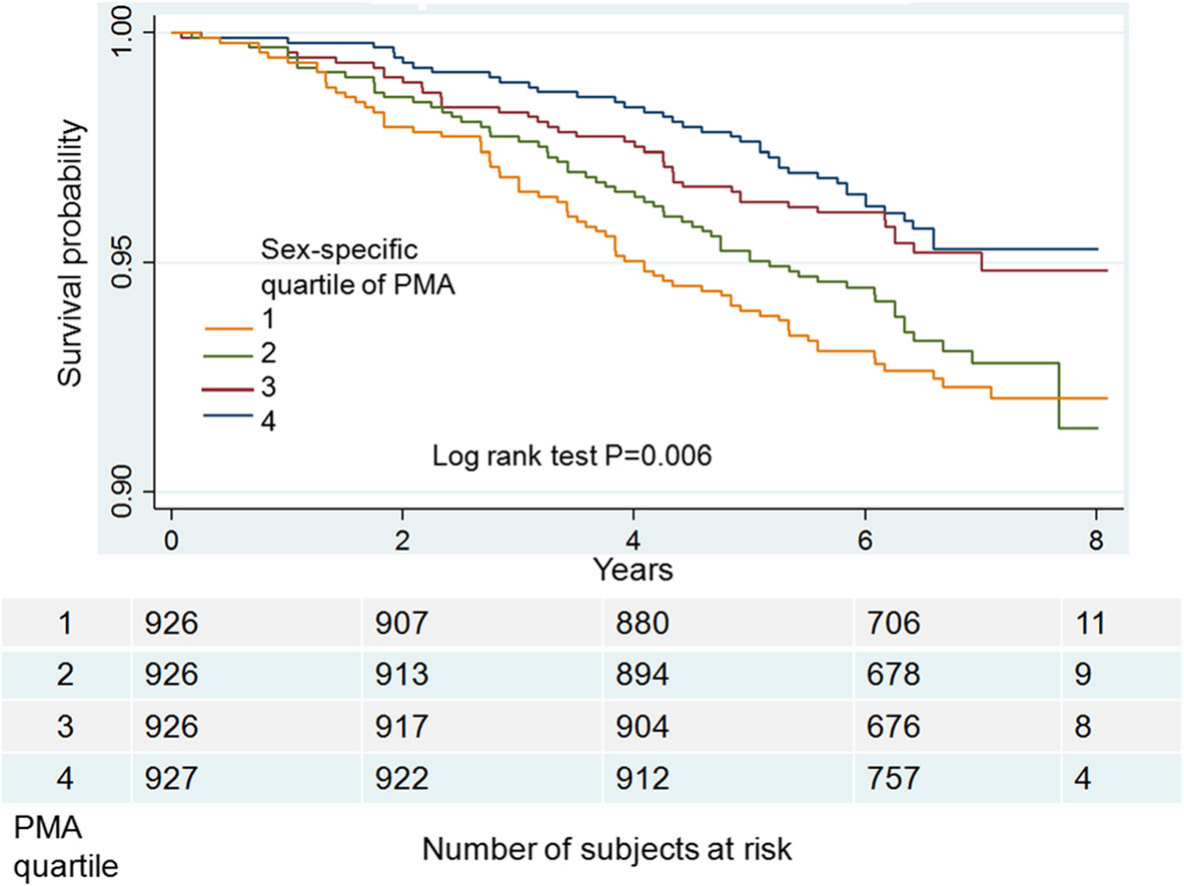
Kaplan-Meier curves for all-cause mortality by sex-specific quartiles of pectoralis muscle area (PMA) in at-risk smokers. The survival probabilities decrease with decreasing PMA quartiles

There were 212 deaths among the 3705 subjects during a median follow-up of 6.5 years. The number of the deaths were higher among at-risk smokers falling in the 2 lowest quartiles of PMA (*N* = 131, 61.7%) and current smokers (*N* = 148, [69.8%]). The Kaplan-Meier plots demonstrate that the probability of survival decreased with decreasing PMA (log-rank test *P* = 0.006) (Fig. 2). There was no relationship between PVMA quartiles and mortality (log-rank test *P* = 0.59) as well as between SAT quartiles and mortality (log-rank test *P* = 0.50). PVMA and SAT were therefore not used in subsequent multivariable models. Multivariable Cox proportional hazard model results using the measures of PMA are shown in Table 2. At-risk smokers in the lowest quartile (quartile 1) and quartile 2 had 85% (Hazard Ratio [HR] 1.85 95% CI [1.14–3.00] and 63% (HR 1.63 [1.04–2.54]) higher risk of death than those in quartile 4, respectively. The results were consistent when the index PMA/height^2^ (i.e., PMA as continuous variable and same covariates as for models using PMA quartiles but height and BMI) was used instead PMA quartiles (HR 0.93 [0.90–0.97] *P* = 0.0002). The association between PMA and mortality was modified by smoking status (*P* = 0.04 for the interaction between PMA and smoking status). Current smokers had a significantly increased risk of death (HR 2.25 [1.25–4.03] *P* = 0.007) while former smokers did not (Table 3). The association between PMA and mortality was not modified by age (*P* = 0.57 for the PMA-age interaction), sex (*P* = 0.44 for the PMA-sex interaction), or race (*P* = 0.50 for the PMA-race interaction).

**Table 2:** Multivariable Cox Regression models for all-cause mortality in all at-risk smokers^a,b^

	HR	95% CI		P
Sex-specific PMA quartile				
4	Ref			
3	1.09	0.70	1.72	0.70
2	1.63	1.04	2.54	0.03
1	1.85	1.14	3.00	0.01

^a^Adjustment for age (years), race (non-Hispanic White, African-American), height (cm), BMI (categories < 20; 20–24.9; 25–30; > 30), pack-years of smoking (per 10), current smoking status (yes/no), mMRC dyspnea score > 1 (yes/no), number of comorbidities (categories, 0; 1–2; ≥3), six-minute walk distance (per 30 m), FEV_1_ (L), log CAC, log %LAA-950, and scanner type was performed

^b^ Model Proportionality Hazard Test, P = 0.13

**Table 3:** Multivariable Cox Regression models for all-cause mortality in at-risk smokers by smoking status^a,b^

	Former smokers			Current smokers		
	HR	95% CI	P	HR	95% CI	P
Sex-specific PMA quartile						
4	Ref			Ref		
3	0.46	0.18–1.13	0.09	1.52	0.89–2.59	0.12
2	0.98	0.44–2.19	0.96	1.94	1.13–3.32	0.02
1	1.22	0.53–2.80	0.64	2.25	1.25–4.03	0.007

^a^Adjustment for age (years), race (non-Hispanic White, African-American), height (cm), BMI (categories < 20; 20–24.9; 25–30; > 30), pack-years of smoking (per 10), mMRC dyspnea score > 1 (yes/no), number of comorbidities (categories, 0; 1–2; ≥3), six-minute walk distance (per 30 m), FEV_1_ (L), log CAC, log %LAA-950, and scanner type was performed

^b^ Model Proportionality Hazard Test, *P* = 0.28 and *P* = 0.13 for current and former smokers, respectively

## Discussion

Low muscle mass is associated with mortality in smokers at risk for developing expiratory airflow obstruction. This association is muscle specific, modified by smoking status, and remains after adjustment for demographics, smoking history, dyspnea, comorbidities, lung function, exercise capacity, emphysema, and coronary artery calcium content.

Prior studies [9–12, 18] and a meta-analysis [43] have examined the association between low muscle mass or sarcopenia and mortality in different populations including community-dwelling subjects, institutionalized individuals, and patients with COPD and cancer. However, we are not aware of studies specifically exploring such an association in smokers without airflow obstruction. Our data indicate that muscle mass but not fat provides prognostic information from body composition in this population. CT scanning can disambiguate the effects of these two components of body composition supporting its utility to further characterize body structure. Multiple studies have now documented the clinical and prognostic consequences of respiratory symptoms and emphysema measured on CT scan in smokers who do not meet the established criteria for COPD [2, 3, 44]. At-risk smokers are also the subject of ongoing therapeutic investigation focused on the improvement of health-related quality of life (NCT02867761 at ClinicalTrials.gov). The aggregate of our work and prior studies further supports the contention that spirometry is insufficient for determining susceptibility to injury to chronic tobacco smoke exposure because it underestimates the presence of pulmonary and extra-pulmonary disease. It is increasingly clear that multimodality assessments of smokers including techniques such as CT imaging are required to more fully determine the clinical manifestations of chronic tobacco smoke exposure.

A second compelling finding in our data was that the association between PMA and death was modified by smoking status. Thus, while among current smokers the association between low muscle mass and all-cause mortality was significant, among former smokers it was not. We believe that the mechanisms that lead to low muscle mass and increased risk of death such as systemic inflammation might be sustained in current compared to former smokers. This difference in the prognostic value of PMA-mortality may also be influenced by differential statistical power as only 1/3 of the deaths occurred among former-smokers. Regardless of the reasons for the difference in risk of death between these groups of smokers, these findings further support smoking cessation as a relevant clinical intervention.

Another interesting observation was the differential associations between the PMA vs. PVMA and an increased risk of death. The PMA but not the PVMA predicted death in the at-risk cohort. The reasons for this are unclear. However, it is possible that PMA is affected by or resilient to different mechanisms of low muscle mass than that of PVMA. Those with preserved PVMA and decreased PMA may be suffering from inactivity, while those with reduced PVMA and PMA are afflicted by a consumptive inflammatory process. This conjecture requires more detailed patients’ characterization, which is not available in COPDGene. An alternate explanation may be found in the distribution of the muscle areas (see Table 1). There may just be a greater biologically plausible range of PMA than PVMA. The relative superiority of PMA for predicting death may not then be directly ascribed to differential muscle wasting but that it is just easier to detect these muscles on CT scan. Perhaps another method to ascertain in-vivo muscle health with higher resolution than CT scan would corroborate this hypothesis. While we have provided compelling observational data linking low muscle mass and mortality in at-risk smokers, we lack the data to provide insight into the mechanisms of low muscle mass in this population. In addition to inflammation and atrophy, studies have also shown aging as a potential explanation for low muscle mass [45, 46]. The current findings are clinically important and pave the way for future mechanistic studies to uncover novel mechanisms of low muscle mass in smokers.

There are limitations to our data that must be acknowledged. COPDGene is a study of heavy smokers and caution must be exercised when extrapolating our results to non-heavy smokers. Further research is needed to confirm our observations in other populations of smokers. Although we lack vital status information in 13% of the subjects, we still had a large cohort with complete data and over 200 events of interest allowing us including a myriad of relevant confounders in our survival models. Additionally, data on mortality cause was not available for this analysis but it is an ongoing effort in the COPDGene Study. Another inherent limitation to observational data is that conclusions cannot be drawn about the response to intervention. While it would seem to be obvious that reversing the low muscle mass in smokers would be beneficial, it is unclear if any intervention from exercise to pharmacologic therapy would improve survival even if the muscle bulk were increased.

## Conclusions

In summary, a single-axial image of the pectoralis muscles seems to be prognostic for patient death in at-risk smokers. Our data provides additional evidence that the extra-pulmonary features evident on CT scan are useful for clinical investigations involving such cohorts of subjects. The PMA measurements are easy to perform and can be obtained from a broad range of CT images such as those being obtained for lung cancer screening or routine clinical care.

## Additional file

Additional file 1 **Figure S1.** Plot of the pectoralis muscle area (PMA) to paravertebral muscle area (PVMA). The relationship between the muscle groups was significant (R^2^ = 0.44, *P* < 0.0001). **Table S1** Baseline characteristics of at-risk smokers by quartile of PVMA (*N* = 3705). (DOCX 207 kb)

## Abbreviations

%LAA-950: Percent of Low-attenuation Areas Less Than −950 Hounsfield Units
6MWD: Six-minute Walk Distance
BMI: Body Mass Index
CAC: Coronary Artery Calcium
CCC: Concordance Correlation Coefficient
COPD: Chronic Obstructive Pulmonary Disease
CT: Computed Tomography
FEV_1_: Forced Expiratory Volume in 1 Second
FEV_1_/FVC: Ratio of Forced Expiratory Volume in 1 second to Forced Vital Capacity
FVC: Forced Vital Capacity
HU: Hounsfield Unit
PMA: Pectoralis Muscle Area
PVMA: Paravertebral Erector Spinae Muscle Area
SAT: Subcutaneous Adipose Tissue
SD: Standard Deviation
