# Supplementary material for: Pectoralis muscle area and mortality in smokers without airflow obstruction
Source: Respir Res. 2018 Apr 10;19:62. doi: 10.1186/s12931-018-0771-6 (PMC5894181; doi:10.1186/s12931-018-0771-6)
Supplement: Supplementary file 1 — Figure S1. Plot of the pectoralis muscle area (PMA) to paravertebral muscle area (PVMA). The relationship between the muscle groups was significant (R2 = 0.44, P < 0.0001). Table S1 Baseline characteristics of at-risk smokers by quartile of PVMA (N = 3705). (DOCX 207 kb) [file 12931_2018_771_MOESM1_ESM.docx]

**Additional File 1 for**

**Pectoralis muscle area and mortality in smokers without airflow obstruction**

^1^Alejandro A Diaz* MD, MPH, ^2^Carlos H Martinez* MD, MPH, ^3^Rola Harmouche PhD, ^1^Thomas P Young BS, ^4^Merry-Lynn McDonald Ph.D, ^3^James C Ross Ph.D, ^2^MeiLan Han MD, ^5^Russell Bowler MD, Ph.D, ^5^Barry Make MD, ^5^Elizabeth A Regan MD, ^1,6^Edwin K Silverman MD, Ph.D, ^5^James Crapo MD, ^7^Aladin M. Boriek, Ph.D, ^8^Gregory L Kinney Ph.D, ^8^John E Hokanson Ph.D, ^3^Raul San Jose Estepar^#^ Ph.D, ^1^George R Washko^#^ MD, MSc

*These authors contributed equally to this work.

^#^Co-senior authors

1. Division of Pulmonary and Critical Care Medicine, Brigham and Women’s Hospital, Harvard Medical School, Boston MA.

2. Division of Pulmonary & Critical Care Medicine, University of Michigan Health System, Ann Arbor MI.

1. Department of Radiology, Brigham and Women’s Hospital, Harvard Medical School, Boston MA.
2. Division of Pulmonary, Allergy and Critical Care Medicine, University of Alabama at Birmingham, AL.
3. Department of Medicine, Division of Pulmonary and Critical Care Medicine, National Jewish Health, Denver CO.
4. Channing Division of Network Medicine, Brigham and Women’s Hospital, Harvard Medical School, Boston MA.
5. Division of Pulmonary and Critical Care Medicine, Baylor College of Medicine, Houston, Texas.
6. Colorado School of Public Health, University of Colorado-Denver, Aurora CO.

**Results**


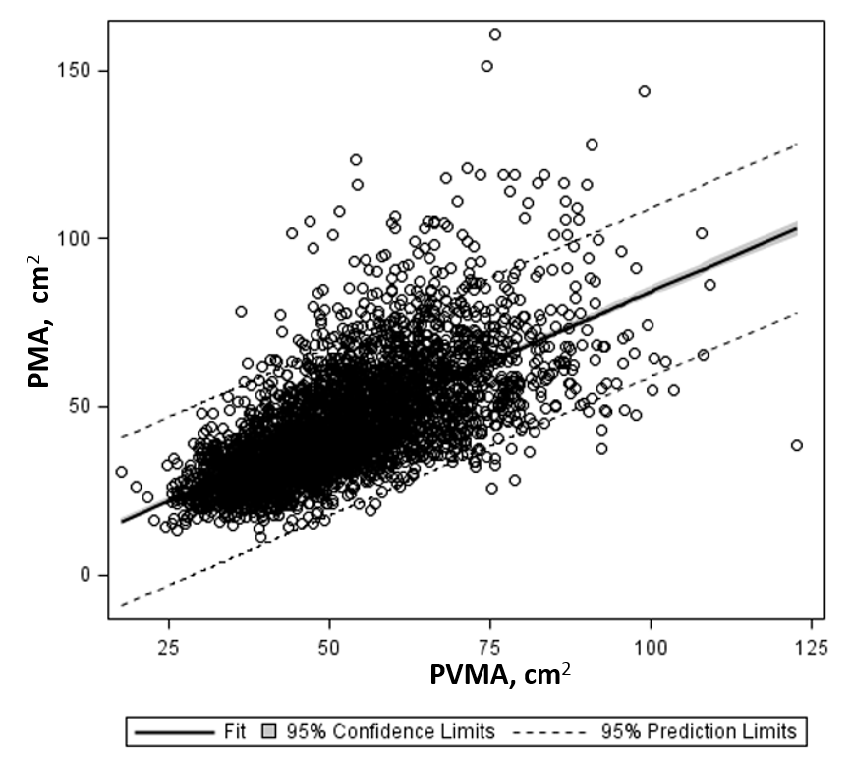


**Figure S1** Plot of the pectoralis muscle area (PMA) to paravertebral muscle area (PVMA). The relationship between the muscle groups was significant (R^2^=0.44, P<0.0001).

**Table S1** Baseline characteristics of at-risk smokers by quartile of PVMA (N=3,705)

|  | **PVMA Quartile*** | | | | | | | |
| --- | --- | --- | --- | --- | --- | --- | --- | --- |
| Variable | 1  (<41.6 cm^2^) | | 2  (41.6-50.5 cm^2^) | | 3  (50.6-60.6 cm^2^) | | 4  (>60.6 cm^2^) | |
| Age, -yrs | 59 | ± 9 | 58 | ± 9 | 57 | ± 8 | 56 | ± 8 |
| Male Sex, -% | 8 |  | 34 |  | 67 |  | 90 |  |
| Non-Hispanic White, -% | 83 |  | 72 |  | 62 |  | 52 |  |
| Height, -cm | 163 | ± 7 | 168 | ± 8 | 173 | ± 8 | 177 | ± 8 |
| BMI, -kg/m2 | 26 | ± 5 | 29 | ± 5 | 30 | ± 6 | 32 | ± 6 |
| Pack Years of Smoking | 36 | ± 19 | 38 | ± 22 | 40 | ± 22 | 40 | ± 21 |
| Current Smoking Status, -% | 46 |  | 54 |  | 58 |  | 61 |  |
| Modified Medical Research Council Dyspnea Score >1, -% | 22 |  | 28 |  | 27 |  | 27 |  |
| No. of Comorbidities*, -% |  |  |  |  |  |  |  |  |
| 0 | 42 |  | 39 |  | 40 |  | 39 |  |
| 1-2 | 49 |  | 50 |  | 48 |  | 49 |  |
| 3 or more | 9 |  | 11 |  | 12 |  | 12 |  |
| FEV_1_, -% predicted | 92 | ± 15 | 93 | ± 15 | 91 | ± 16 | 91 | ± 15 |
| FVC, -% predicted | 92 | ± 14 | 92 | ± 15 | 91 | ± 15 | 91 | ± 15 |
| Six-minute walk distance, -m | 456 | ± 100 | 450 | ± 106 | 444 | ± 117 | 456 | ± 112 |
| PMA, -cm^2^ | 30.3 | ± 7.8 | 38.7 | ± 11.5 | 48.3 | ± 13.8 | 59.8 | ± 17.4 |
| PVMA, -cm^2^ | 36.1 | ± 3.9 | 46.0 | ± 2.6 | 55.3 | ± 2.9 | 70.6 | ± 8.8 |
| SAT, -cm^2^ | 62.7 | ± 31.1 | 67.8 | ± 38.0 | 61.4 | ± 43.1 | 55.8 | ± 34.4 |
| %LAA-950 on CT scans, -% | 1.8 | ± 2.7 | 1.9 | ± 2.6 | 2.0 | ± 2.5 | 2.0 | ± 2.3 |
| CAC score | 74 | ± 192 | 93 | ± 276 | 130 | ± 291 | 158 | ± 372 |

Data are presented as number, proportion (percentage), and mean ± SD.

*PVMA quartiles are not sex specific.

Missing data for SAT, 44.
